# Teacher Personality Predicts Emotional Well-Being and Academic Achievement in Students with Specific Learning Disorders

**DOI:** 10.3390/children12060764

**Published:** 2025-06-13

**Authors:** Wanqin Yu, Olivia F. Ward, Brianna Paquette, Sylvie Mrug, Caroline G. Richter

**Affiliations:** Department of Psychology, The University of Alabama at Birmingham, 1720 2nd Ave S, Birmingham, AL 35294, USA; yuw@uab.edu (W.Y.); oward@uab.edu (O.F.W.); bristein@uab.edu (B.P.); sylva@uab.edu (S.M.)

**Keywords:** teacher personality, big five traits, emotional well-being, academic achievement, specific learning disorders, multilevel modeling

## Abstract

**Background/Objectives:** Students with specific learning disorders (SLDs) are at increased risk for emotional and academic difficulties. While teacher characteristics can influence student outcomes, few studies have examined the role of teacher personality in supporting students with SLDs. This study investigated whether teacher personality traits predicted student emotional well-being and academic achievement in a school-based intervention context. **Methods**: Participants were 64 students with SLDs (Mage = 13.28) nested within 21 teachers. Students completed measures of emotional well-being at baseline and post-intervention, and grade point average (GPA) was obtained from school records at the end of the school year. Teachers completed the Big Five Inventory mid-intervention. Two-level multilevel models were conducted in Mplus using maximum likelihood estimation with robust standard errors (MLR). The models controlled for student and teacher demographics, baseline emotional well-being, and the intervention group. Missing data were addressed using full information maximum likelihood (FIML). **Results**: Teacher female sex, higher neuroticism, and lower teaching experience were associated with higher student emotional well-being post-intervention. Follow-up analyses confirmed that teacher sex, neuroticism, and conscientiousness each explained substantial between-teacher variance. In the GPA model, student sex and teacher openness were significant predictors, with female students and students taught by more open teachers earning higher GPAs. **Conclusions**: Teacher personality traits, specifically neuroticism, conscientiousness, and openness, were associated with emotional and academic outcomes among students with SLDs. The findings highlight the importance of considering teacher characteristics in designing school-based interventions to support the development of learners with SLDs or other neurodevelopmental disorders.

## 1. Introduction

Because students spend a significant portion of their lives in school, teachers are key figures in promoting both their emotional well-being and academic achievement [1,2]. Given the pivotal role teachers play in children’s lives, understanding teacher-related factors, such as personality, may inform how education systems tailor support to foster positive student outcomes. This is particularly critical for students who are at heightened risk for school-related challenges, such as those with specific learning disorders (SLDs) [3,4]. The present study examined how teacher personality traits relate to emotional well-being and academic achievement in students with SLDs.

SLDs are a group of neurodevelopmental disorders characterized by difficulties in academic domains such as reading, comprehension, spelling, written expression, and/or mathematics [5]. SLDs are among the most prevalent disorders in school-aged children, with rates estimated between 5% and 15% [6,7]. These disorders can negatively impact not only students’ academic performance but also their social and emotional development [3,8,9]. Because many students with SLDs face ongoing academic struggles and repeated failure in school, they are more likely to experience frustration, lowered self-esteem, and negative perceptions of their abilities. These experiences contribute to elevated levels of internalizing problems, such as anxiety, depression, and general psychological distress, that are widely reported in this population. In particular, SLDs have been identified as a risk factor for internalizing problems that contribute to poorer emotional well-being [4,9,10,11,12]. These challenges often persist into adulthood and are associated with lower rates of college completion, underemployment, and increased interaction with the criminal justice system [13]. Nonetheless, many students with SLDs go on to achieve success and often attribute their progress to positive relationships with teachers, peers, and mentors [14,15]. This aligns with a cumulative risk and resilience perspective, which emphasizes that the presence of protective factors, such as supportive relationships, can buffer the negative effects of learning difficulties [16].

Insights from attachment theory support the importance of teacher–student relationships in shaping emotional outcomes. Attachment theory suggests that behaviors aimed at establishing closeness and contact with adult figures (e.g., parents) are maintained to aid in meeting the child’s needs [17]. Teacher–student relationships mimic parent–child relationships in that they both involve care, teaching, and discipline [18]. As such, teachers can become a secure base as children navigate the world [19]. Previous studies have found that greater perceived teacher support positively predicts students’ abilities to navigate academic challenges [20], greater student academic efficacy [21], and greater student academic achievement [22]. Given the integral role teachers play in students’ lives, it is critical to understand how teacher characteristics, such as personality traits, may influence outcomes for students with SLDs.

### 1.1. Teacher Personality in an Educational Context

While much of the literature has focused on the instructional or relational roles teachers play, emerging research highlights the importance of teacher personality in shaping students’ school experiences. Personality theory, particularly the Five-Factor Model, offers a valuable framework for understanding how individual differences among teachers may influence educational processes [23]. In particular, traits such as conscientiousness, emotional stability, and extraversion are identified as especially relevant for effective teaching, with conscientiousness supporting structured and goal-oriented classroom management, emotional stability helping teachers manage negative emotions, and extraversion contributing to classroom engagement and positive teacher–student interactions [23].

Meta-analytic evidence underscores the relevance of teacher personality in relation to teacher effectiveness, a construct closely tied to classroom organization, instructional quality, and professional competence [24]. Among the Big Five traits, extraversion demonstrates the strongest overall association with teacher effectiveness, followed by conscientiousness, emotional stability, and openness. These traits are consistently linked to positive teaching behaviors across multiple rating sources. Agreeableness, in contrast, is not significantly associated with teacher effectiveness; it is also not consistently linked to key instructional or classroom outcomes across studies [24]. In addition, researchers have found that teachers with a “well-adjusted” personality profile, characterized by high conscientiousness, extraversion, agreeableness, and openness, and low neuroticism, report greater self-efficacy, engagement with students, and job satisfaction [25]. These qualities may help foster emotionally supportive and structured classrooms that are particularly beneficial for students with SLDs. Indeed, research has shown that teacher self-efficacy plays a central role in shaping instructional quality, classroom climate, and student academic adjustment [26].

### 1.2. Teacher Personality and Student Emotional Well-Being

Many individuals with SLDs experience elevated levels of anxiety, depression, poor mental health, and lower self-esteem in addition to their academic challenges [12]. These internalizing difficulties can further hinder academic success [27,28], creating a negative cycle that compounds the challenges already faced by students with SLDs. However, not all students with SLDs report poor emotional well-being; many demonstrate positive adjustment despite risk factors associated with their SLD [15,29]. Teacher support, in particular, has been identified as a key protective factor in this process [30,31]. Among typically developing students, positive student–teacher relationships are associated with greater student emotional well-being [32]. When compared to support from caregivers and peers, teacher support has been shown to demonstrate the strongest association with students’ emotional well-being [33]. Therefore, understanding teacher-related factors is critical for fostering emotional well-being in students with and without SLDs.

Research suggests that positive student–teacher relationships are characterized by high levels of closeness and warmth and low levels of conflict [34,35]. Therefore, teachers who are able to foster closeness and minimize conflict may be better positioned to support students with SLDs. Additionally, students who perceive greater teacher support report higher levels of emotional well-being, both in general school populations [33] and among students with SLDs [30]. As such, teacher personality traits, such as agreeableness, extraversion, conscientiousness, and openness, that facilitate supportive, low-conflict relationships may be particularly important for promoting emotional well-being in students with SLDs.

### 1.3. Teacher Personality and Student Academic Achievement

In addition to well-being, teacher support may also contribute to improved academic outcomes. For example, emotional support from homeroom teachers has been shown to positively predict class-level academic achievement, in part by fostering a more emotionally positive classroom climate [36,37]. Similarly, teacher emotional support has been linked to improved student math performance, with effects mediated by students’ academic self-efficacy and behavioral engagement [38]. Extensive meta-analytic evidence supports the link between teacher–student relationships and academic achievement, including GPA. Positive affective relationships have been shown to promote school engagement and learning outcomes [39], while emotionally supportive classroom climates have been associated with higher academic performance and reduced socioemotional distress [37]. These findings suggest that teacher traits promoting supportive, emotionally responsive interactions may be beneficial not only for students’ well-being but also their academic success.

Among students without academic difficulties, positive student–teacher relationships are associated with higher academic achievement [40]. Students with SLDs tend to demonstrate lower academic performance than their typically developing peers, as their learning difficulties often affect information processing and subject-specific skills [41]. However, research suggests that with appropriate instruction, accommodations, and contextual supports, students with SLDs can achieve academic outcomes comparable to their peers [6,16,42]. These findings underscore the importance of identifying factors that influence academic achievement in this population. One such factor may be teachers’ personality traits.

Research has yielded mixed findings regarding the impact of teachers’ personality traits on student outcomes. Some studies suggest that traits such as extraversion, conscientiousness, agreeableness, and openness to experience are associated with greater student academic achievement in general education settings [43,44,45,46,47]. In contrast, other research has shown that teacher personality traits are more predictive of subjective measures of teaching effectiveness than of students’ objective academic achievement [24,48]. These findings suggest that while certain teacher traits may help students feel more supported, this does not always translate into measurable academic gains. Notably, most of this research has been conducted with general student populations, leaving open questions about whether these patterns hold for students with SLDs. Because students with SLDs often benefit from individualized and sustained teacher support, it is important to examine how teacher personality traits may influence their academic achievement.

### 1.4. Current Study

The present study aimed to examine the associations between teacher personality traits and student outcomes, specifically, emotional well-being and academic achievement, among adolescents with SLDs. Demographic variables, including the age and sex of both students and teachers, and teachers’ years of teaching experience, were included as covariates due to their previously documented associations with student outcomes [49,50]. Based on previous studies [23,24,25,44,46,47], it was hypothesized that teacher traits of openness, conscientiousness, extraversion, and agreeableness would positively predict student academic achievement and emotional well-being over time, controlling for baseline characteristics. In contrast, teacher neuroticism was expected to negatively predict both student outcomes.

## 2. Methods

### 2.1. Procedure

Data for the current study were collected as part of a larger longitudinal project evaluating the biopsychosocial outcomes of mindfulness-based instruction on adolescents with SLDs. Participants were randomly assigned to one of two social and emotional learning (SEL) curricula: a mindfulness-based SEL curriculum (MindUP) [51], or “Normal Isn’t Real” (NIR), which aimed to promote neurodiversity awareness [52]. Both programs were administered by the teachers for 17 weeks. This study was publicly preregistered on ClinicalTrials.gov, https://clinicaltrials.gov/study/NCT05787483 (accessed on 1 June 2025), including the research design and planned analyses, prior to data collection and analysis. The study protocol was reviewed and approved by the university’s Institutional Review Board. Parents or legal guardians of all participants provided written informed consent, and participants provided written assent. Children completed the measures at their school, which were administered by trained doctoral students or research assistants and scored according to standardized procedures. For this study, surveys completed at specific timepoints were used: students completed surveys at baseline and at post-intervention (week 18), while teachers completed surveys at the intervention midpoint (week 9).

### 2.2. Participants

The initial sample included 68 students nested within 22 teachers. One teacher was excluded due to missing data on all Big Five personality traits, resulting in the removal of their three corresponding students. Additionally, one student was excluded prior to analysis after being identified as a statistical outlier, with a standardized residual of –2.70 and a disproportionate influence on residual normality. The final analytic sample consisted of 64 students nested within 21 teachers.

Participants included in this study were 64 students enrolled in Grades 5 through 12 at a private school in Alabama serving children and adolescents with learning disorders. Students ranged in age from 10 to 19 years (*M* = 13.28, *SD* = 2.24), with equal distribution by sex (50% female, 50% male) and a near-even split between intervention groups (46.9% MindUP, 53.1% NIR). Students in the MindUP group did not differ significantly from those in the NIR group in terms of age, *t*(62) = 0.38, *p* = 0.703; sex, *t*(62) = 0.49, *p* = 0.623; or baseline emotional well-being, *t*(61) = 0.47, *p* = 0.643.

A total of 21 teachers were included in this study, each responsible for an average of 4.39 students (*SD* = 0.89). Teachers ranged in age from 26 to 62 years (*M* = 39.05, *SD* = 11.86), with one participant missing age data. Most teachers were female (66.7%), and all identified as White and non-Hispanic. All teachers held at least a bachelor’s degree, and 42.9% reported more than 10 years of teaching experience. Teachers in the MindUP and NIR groups did not differ significantly on sex, *t*(19) = 1.56, *p* = 0.135; years of teaching experience, *t*(19) = −0.73, *p* = 0.473; or any of the Big Five personality traits (all *p*s > 0.05).

### 2.3. Measures

#### 2.3.1. Big Five Inventory-10 (BFI-10)

Teachers’ personality traits were assessed using the BFI-10 [53], a brief 10-item self-report inventory designed to measure the Big Five personality domains: openness, conscientiousness, extraversion, agreeableness, and neuroticism. Each domain is assessed with two items. Teachers rated items on a 5-point Likert scale ranging from 1 (disagree strongly) to 5 (agree strongly), and trait scores were computed by averaging the two items corresponding to each domain. The BFI-10 has demonstrated strong part–whole correlations with the BFI-44 scales (*r*s = 0.74–0.89), acceptable test–retest reliability over 6–8 weeks (*r*s = 0.65–0.87), and good convergent and external validity [53]. Although internal consistency estimates were not reported in the original validation study, consistent with psychometric literature cautioning against the use of Cronbach’s alpha for two-item scales [54], recent large-sample research has provided strong support for the BFI-10′s reliability. In a nationally representative Brazilian sample (*n* = 3565), the BFI-10 showed good reliability for all five traits, with omega coefficients ranging from 0.72 to 0.88 [55]. However, in the present sample (*n* = 21), only the extraversion subscale demonstrated acceptable internal consistency (*α* = 0.62). The remaining subscales showed poor internal consistency: openness (*α* = 0.52), neuroticism (*α* = 0.43), agreeableness (*α* = 0.36), and conscientiousness (*α* = 0.11), likely reflecting both the scale’s brevity and the small sample size.

#### 2.3.2. Brief Multidimensional Students’ Life Satisfaction Scale (BMSLSS-PTPB)

Students’ emotional well-being was assessed using the Brief Multidimensional Students’ Life Satisfaction Scale–Peabody Treatment Progress Battery version (BMSLSS–PTPB) [56]. This 6-item self-report measure was administered at baseline and post-intervention. Items were rated on a 5-point Likert scale ranging from 0 (very dissatisfied) to 5 (very satisfied). One item assesses overall life satisfaction, while the remaining five items assess satisfaction across specific life domains: family life, friendships, school experience, self-perception, and living environment. Scores were averaged to create a total emotional well-being score, with higher scores indicating greater life satisfaction. Internal consistency in the current sample was good at both timepoints, with Cronbach’s alpha increasing from baseline (*α* = 0.80) to post-intervention (*α* = 0.88). This aligns with, and slightly exceeds, the previously reported reliability in clinical samples (*α* = 0.77) [57] and falls within the range observed in normative school-based samples (*α* = 0.76–0.85) [58].

#### 2.3.3. Grade Point Average (GPA)

GPA was utilized to measure students’ academic achievement. Students’ GPA was reported by the school on a scale ranging from 0.0 to 4.0; grades of A (3.1–4.0) were reported as 4.0, grades of B (2.1–3.0) as 3.0, grades of C (1.1–2.0) as 2.0, grades of D (0.1–1.0) as 1.0, and grades of F as 0.0. Students’ end-of-year GPA was calculated as the arithmetic mean of first-semester letter grades and second-semester letter grades. The courses considered in the calculation were math, English, history, science, and health. Students’ school-reported GPA was obtained at the end of the school year, which corresponds to a few months post-intervention. Of the twenty-one teachers, five taught math, five taught English, three taught history, three taught science, and five were reading intervention teachers.

#### 2.3.4. Demographics Survey

Student demographic information was collected through parent-reported data. Teacher demographic information was collected through self-reported surveys, including race, sex, years of teaching experience, and highest level of education. Teachers selected the label that best described their highest level of education from the following list: high school diploma or General Equivalency Diploma (GED), associate’s degree, bachelor’s degree, some graduate coursework (but no degree), master’s degree, education specialist or professional diploma (post-master’s), or doctorate.

### 2.4. Statistical Analyses

Missing data were examined, and cases with any vs. no missing data were compared on all variables using independent samples t-tests for continuous variables and chi-square tests of independence for categorical variables. Descriptive statistics were examined. Pearson’s bivariate correlations were conducted among all primary study variables, evaluating three sets of associations among (1) student-level variables, (2) teacher-level variables, and (3) cross-level correlations between student and teacher characteristics.

Assumptions of multilevel modeling (MLM) were evaluated in SPSS (Version 30) prior to model estimation in Mplus. Restricted maximum likelihood (REML) was used during assumptions testing, as it provides unbiased estimates of variance components. Intraclass correlation coefficients (ICCs) were calculated using random intercept models to assess the proportion of variance attributable to the teacher level. The assumption of homoscedasticity was examined via residual plots and the Breusch–Pagan test. Normality of residuals was tested with Kolmogorov–Smirnov and Shapiro–Wilk tests. The linearity between continuous predictors and outcomes was evaluated with bivariate scatterplots. Finally, the absence of multicollinearity was verified with the variance inflation factor (VIF) and tolerance.

Main models were estimated in Mplus Version 8.1 using full information maximum likelihood (FIML) with the robust maximum likelihood (MLR) estimator to appropriately handle missing data and assumption violations. Two multilevel models with two levels were estimated to predict students’ emotional well-being and GPA, respectively. In both models, students (Level 1) were nested within teachers (Level 2), and random intercepts were specified to account for this clustering. In the first model predicting students’ post-intervention emotional well-being, student-level (within) predictors included age, sex at birth, intervention group, and baseline emotional well-being. Teacher-level (between) predictors included sex, years of teaching experience, and Big Five personality traits (extraversion, agreeableness, conscientiousness, neuroticism, and openness). In the second model predicting students’ end-of-school-year GPA, student-level predictors were age, sex at birth, and intervention group, while teacher-level predictors were identical to those in the emotional well-being model. All continuous predictors were centered prior to analysis to facilitate interpretation of the results: student-level variables were group-mean-centered, and teacher-level variables were grand-mean-centered. Categorical predictors were dummy coded.

## 3. Results

### 3.1. Preliminary Analyses

Among the 64 students included in the analytic sample, 10.9% (*n* = 7) had missing data on emotional well-being variables used in the multilevel models. Specifically, six students were missing post-intervention emotional well-being scores, and one was missing baseline emotional well-being. Independent samples *t*-tests were conducted to examine whether this missingness was associated with any study variables. There was no significant difference in post-intervention emotional well-being between those with and without missing data, *t*(56) = 0.32, *p* = 0.751. A corrected *t*-test was used for end-of-school-year GPA due to unequal variances, which also revealed no significant difference, *t*(6.50) = 1.46, *p* = 0.190. Additionally, missingness was unrelated to all student-level covariates, including age, sex, intervention group, and baseline emotional well-being (all *p*s > 0.05), supporting the assumption that data were missing at random (MAR).

At the teacher level, there was a significant difference in years of teaching experience, *t*(55.35) = –5.22, *p* < 0.001, such that students with missing data had teachers with more experience (*M* = 9.57, *SD* = 0.54) than those with complete data (*M* = 7.21, *SD* = 3.06). No significant differences were found for teacher sex, *t*(8.65) = –1.44, *p* = 0.185; extraversion, *t*(62) = 1.86, *p* = 0.068; agreeableness, *t*(62) = 0.19, *p* = 0.850; conscientiousness, *t*(62) = –1.36, *p* = 0.178; neuroticism, *t*(62) = 0.97, *p* = 0.335; or openness, *t*(62) = 1.15, *p* = 0.254.

Descriptives (see Table 1) showed that students reported moderately high levels of post-intervention emotional well-being (*M* = 4.08, *SD* = 0.77) on a scale ranging from 0 to 5. Their academic performance, assessed via school-reported GPA, averaged 3.70 (*SD* = 0.35). Teachers had an average of 7.81 years of teaching experience (*SD* = 2.89). On average, teachers scored slightly above the midpoint (i.e., neither agree nor disagree) on the Big Five personality traits of agreeableness (*M* = 3.24, *SD* = 0.87), conscientiousness (*M* = 3.93, *SD* = 0.75), neuroticism (*M* = 3.38, *SD* = 0.91), and openness (*M* = 3.83, *SD* = 0.93), but slightly below the midpoint on extraversion (*M* = 2.52, *SD* = 1.03), based on a scale ranging from 1 to 5.

Pearson’s bivariate correlations (see Table 1) revealed that at the student level, older students reported significantly lower emotional well-being at both timepoints, *r* = –0.37, *p* = 0.003 (baseline), and *r* = –0.38, *p* = 0.003 (post-intervention). Baseline emotional well-being was strongly and positively associated with post-intervention emotional well-being, *r* = 0.78, *p* < 0.001.

Correlations among teacher-level variables were computed using a dataset aggregated at the teacher level to preserve independence. More years of teaching experience were significantly associated with lower extraversion, *r* = –0.47, *p* = 0.032. Female teachers reported significantly lower levels of openness, *r* = –0.47, *p* = 0.033. Openness was also negatively associated with conscientiousness, *r* = –0.45, *p* = 0.040.

Several significant correlations emerged between student-level and teacher-level variables. Older students were more likely to have male teachers, *r* = –0.49, *p* < 0.001, and teachers who scored lower on agreeableness, *r* = –0.44, *p* < 0.001. Students in the MindUP group were more likely to have male teachers, *r* = –0.38, *p* = 0.002, and teachers who scored higher on openness, *r* = 0.32, *p* = 0.011. Additionally, students with female teachers reported higher levels of emotional well-being at both timepoints, *r* = 0.26, *p* = 0.037 (baseline), and *r* = 0.40, *p* = 0.002 (post-intervention).

Assumption testing indicated that for emotional well-being, the ICC was 0.181, and for GPA, the ICC was 0.054, indicating that 18.1% and 5.4% of the variance, respectively, were due to differences between teachers. Both values fell within the range of 0.05 to 0.25, commonly observed in educational research, supporting the use of MLM to account for the nested structure of students within teachers [59].

Residual plots suggested homoscedasticity for emotional well-being but heteroscedasticity for GPA. This violation was confirmed by a significant Breusch–Pagan test for GPA, *F*(1, 62) = 29.80, *p* < 0.001. Attempts to correct the heteroscedasticity through data transformations were unsuccessful. As a result, both models were estimated using the MLR estimator in Mplus to account for this violation. The normality of residuals was supported for emotional well-being, as indicated by non-significant results from both the Kolmogorov–Smirnov test, *p* = 0.200, and the Shapiro–Wilk test, *p* = 0.645. For GPA, residual normality was less clear, with the Kolmogorov–Smirnov test indicating a significant deviation from normality, *p* = 0.013, while the Shapiro–Wilk test was not significant, *p* = 0.060. Minor non-normality in GPA residuals was addressed using robust maximum likelihood estimation. Visual inspection confirmed linear relationships between continuous predictors and outcomes. Multicollinearity diagnostics indicated no concerns, with all VIF values below 2.40 and tolerance values above 0.40.

### 3.2. Main Analyses

All 64 students had complete data for the GPA full model, while only 57 students had complete data for the emotional well-being full model. Both models were estimated in Mplus using the MLR estimator with FIML to handle missing data. The emotional well-being model has an average cluster size of 2.71 and an ICC of 0.380, while the academic achievement model has an average cluster size of 3.05 and an ICC of 0.114.

#### 3.2.1. Emotional Well-Being Model

**Figure 1:**
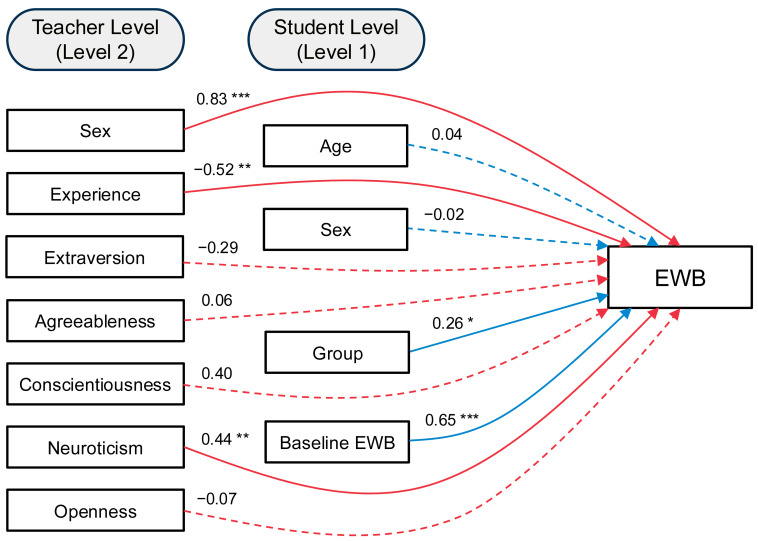
**Graphical representation of MLM predicting post-intervention EWB.** *Note.* MLM = multilevel model; EWB = emotional well-being; Experience = years of teaching experience. Sex was coded as 0 = male, 1 = female. Group was coded as 0 = Normal Isn’t Real, 1 = MindUP. *n* = 57 students with complete data. All coefficients are standardized. * *p* < 0.05. ** *p* < 0.01. *** *p* < 0.001.

A two-level multilevel model was estimated to examine predictors of students’ post-intervention emotional well-being, accounting for the nesting of students (Level 1) within teachers (Level 2). Complete model results, including unstandardized estimates, standard errors, and significance levels, are presented in Table 2, along with standardized coefficients to facilitate interpretation of effect sizes (see Figure 1). At the student level, higher baseline emotional well-being significantly predicted greater post-intervention emotional well-being (*b* = 0.70, *SE* = 0.10, *p* < 0.001). Students assigned to the MindUP group also reported significantly higher emotional well-being compared to those in the NIR group (*b* = 0.33, *SE* = 0.16, *p* = 0.034). Student age and sex were not significant predictors (*b* = 0.03, *SE* = 0.08, *p* = 0.708; *b* = –0.02, *SE* = 0.12, *p* = 0.860, respectively).

At the teacher level, students taught by female teachers reported significantly higher emotional well-being (*b* = 0.89, *SE* = 0.19, *p* < 0.001). Higher teacher neuroticism was positively associated with student emotional well-being (*b* = 0.25, *SE* = 0.08, *p* = 0.003), whereas more years of teaching experience was negatively associated with student emotional well-being (*b* = –0.09, *SE* = 0.03, *p* = 0.001). Teacher conscientiousness was not a significant predictor (*b* = 0.28, *SE* = 0.14, *p* = 0.055). Teacher extraversion, agreeableness, and openness were all not significant (*p*s > 0.10). While these non-significant results may reflect true null effects, it is also possible that the small number of teacher-level observations limited our ability to detect associations of modest size. After accounting for covariates and predictors, the residual variance at the student level was 0.22 (*SE* = 0.06, *p* < 0.001), and the residual between-teacher variance was negligible and non-significant (0.002, *SE* = 0.06, *p* = 0.978). The model explained 47.4% of the variance in emotional well-being at the student level (*p* < 0.001) and 99.3% at the teacher level (*p* < 0.001).

Given the unusually high R-square at the between level, several follow-up models were tested to examine the plausibility of this value. First, individual teacher-level predictors were entered separately into four additional models. When teacher sex, neuroticism, and conscientiousness were tested as individual predictors while controlling for covariates, each emerged as significant. Teacher sex alone significantly predicted emotional well-being (*b* = 0.75, *SE* = 0.28, *p* = 0.006) and explained 53.8% of the between-level variance (*p* = 0.005). Teacher neuroticism was a significant positive predictor (*b* = 0.19, *SE* = 0.08, *p* = 0.012), accounting for 70.4% of the between-level variance (*p* = 0.001). Teacher conscientiousness also significantly predicted emotional well-being (*b* = 0.32, *SE* = 0.15, *p* = 0.029), explaining 79.8% of the between-level variance (*p* < 0.001). In contrast, teaching experience was not a significant predictor (*b* = 0.03, *SE* = 0.03, *p* = 0.376) and explained only 2.7% of the between-level variance (*p* = 0.666). These findings suggest that teacher sex, neuroticism, and conscientiousness may be driving a large portion of the between-level variance, and that the high R-square observed in the full model likely reflects a combination of overlapping contributions from multiple teacher-level characteristics.

#### 3.2.2. Academic Achievement Model

**Figure 2:**
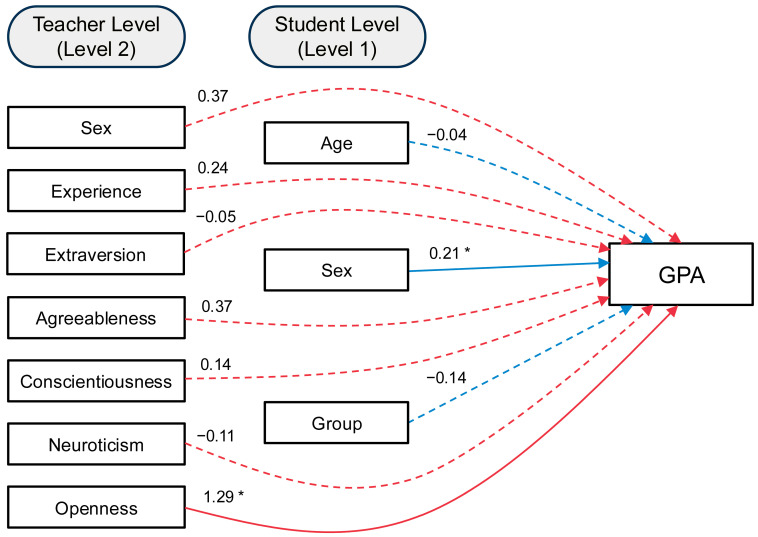
**Graphical representation of MLM predicting end-of-school-year GPA.** *Note*. MLM = multilevel model; GPA = grade point average; Experience = years of teaching experience. Sex was coded as 0 = male, 1 = female. Group was coded as 0 = Normal Isn’t Real, 1 = MindUP. *n* = 64 students with complete data. All coefficients are standardized. * *p* < 0.05.

A second two-level multilevel model examined predictors of students’ academic achievement (end-of-school-year GPA). Complete model results, including unstandardized estimates, standard errors, and significance levels, are presented in Table 3, along with standardized coefficients to facilitate interpretation of effect sizes (see Figure 2). At the student level, sex was a significant predictor, with school records showing that girls achieved a higher GPA than boys (*b* = 0.14, *SE* = 0.07, *p* = 0.038). Student age and intervention group were not significantly associated with GPA (*b* = –0.02, *SE* = 0.05, *p* = 0.772; *b* = –0.10, *SE* = 0.09, *p* = 0.256, respectively). At the teacher level, greater teacher openness significantly predicted higher GPA (*b* = 0.17, *SE* = 0.07, *p* = 0.022). No other teacher-level predictors reached significance, including teacher sex (*b* = 0.09, *SE* = 0.11, *p* = 0.393), teaching experience (*b* = 0.01, *SE* = 0.02, *p* = 0.560), and the remaining personality traits (all *p*s > 0.10).

After accounting for covariates and predictors, the residual variance in GPA remained significant at the student level (*b* = 0.10, *SE* = 0.02, *p* < 0.001), while the between-teacher residual variance was negligible and non-significant (*b* = 0.00, *SE* = 0.01, *p* = 0.967). The model explained 7.3% of the variance in GPA at the student level (*p* = 0.196) and 96.9% at the teacher level (*p* = 0.190); however, neither R-square value reached statistical significance. To examine the robustness of the significant effect for openness, a follow-up model tested openness as the only teacher-level personality predictor while controlling for teacher sex and teaching experience. In this model, teacher openness remained a significant predictor of student GPA (*b* = 0.13, *SE* = 0.06, *p* = 0.033), suggesting this effect is not dependent on other personality traits in the model. However, the between-level R-square was not significant (0.961, *p* = 0.353), indicating that substantial unexplained variance remained.

## 4. Discussion

### 4.1. Summary of Findings

The present study investigated how teacher personality traits influence emotional well-being and academic achievement among students with SLDs, a population often underrepresented in research on teacher–student dynamics [15,16,29]. While previous studies have examined the relationship between teacher personality and student outcomes in general populations [23,24,44,46,47,48], this study extends the literature by focusing on students with SLDs and using a multilevel framework to capture teacher-level effects.

Findings partially supported the initial hypotheses and offered nuanced insights into the influence of teacher personality traits on student outcomes [23]. Contrary to expectations, higher teacher neuroticism significantly predicted greater student emotional well-being following the intervention [23,24,25,44,46]. Although this finding diverges from prior literature linking teacher depressive symptoms to poorer student achievement [60], it may reflect the benefits of emotional sensitivity. Neuroticism reflects a tendency toward emotional sensitivity, anxiety, and self-consciousness [61]. While often associated with negative outcomes, certain facets of neuroticism, such as heightened emotional awareness, may enhance a teacher’s ability to recognize and respond to students’ emotional needs. Teachers higher in neuroticism may be more emotionally attuned and better able to create classroom environments that are validating and responsive to students’ affective experiences. This interpretation aligns with research suggesting that teachers who develop greater emotional awareness and regulation through mindfulness training can foster psychologically safe and supportive classroom environments [62]. Notably, of the 21 teachers, 9 scored at or below the midpoint and 12 scored above it, indicating that neuroticism levels in this sample were generally moderate rather than extreme. It is possible that moderate levels of neuroticism are adaptive, whereas higher levels may produce different effects. Future research should explore potential nonlinear (e.g., U-shaped) associations between teacher neuroticism and student well-being.

It is also important to consider the non-significant findings in light of this study’s statistical power. Although traits such as agreeableness and extraversion have been linked to student outcomes in prior studies [23,24,44,46,47], these traits did not reach significance in our models. This may reflect a true lack of association in this context, or it may be due to the modest number of clusters, which reduces power to detect smaller effects. By reporting confidence intervals alongside estimates, we aim to transparently communicate the level of uncertainty around these findings and encourage cautious interpretation.

Teacher conscientiousness did not significantly predict student emotional well-being in the full model but did emerge as a significant predictor when examined in isolation. This finding supports prior research suggesting that conscientious teachers, who tend to be organized, responsible, and attentive, are more likely to demonstrate effective classroom management, greater support for students, and adaptability to diverse learning needs [23,24,25,48]. These qualities may function as protective factors for students with SLDs, promoting their socioemotional resilience [29,30,31]. In particular, teacher conscientiousness may contribute to the creation of structured yet flexible learning environments that are especially beneficial for students requiring additional support [23,24].

As hypothesized, greater teacher openness significantly predicted higher student GPA post-intervention [23,24,25,44,46,47]. This finding aligns with prior work indicating that more open teachers, who are often imaginative, flexible, and intellectually curious, are more likely to implement adaptive and inclusive instructional strategies [23,24,61,63]. Such characteristics may be especially beneficial for students with SLDs, who are known to benefit from creative, individualized, and differentiated instructional approaches [42]. In contrast, teacher agreeableness and extraversion were not significantly associated with student outcomes in this study. This aligns with prior research indicating that while these personality traits may enhance students’ perceptions of teacher support and foster greater self-efficacy, they do not directly predict objective measures such as academic achievement [24,48]. These findings suggest that although interpersonal warmth and sociability are important for relational aspects of teaching [23], they may not be sufficient to influence academic or emotional outcomes in students with SLDs.

Beyond personality traits, several student and teacher characteristics also contributed to post-intervention outcomes. Students with higher baseline emotional well-being showed higher post-intervention emotional well-being, as expected. Participation in the MindUP intervention was also associated with increased emotional well-being. This finding is in line with previous research demonstrating the positive effects of MindUP and other SEL programs on students’ emotional well-being [64,65], highlighting the program’s potential value for supporting students with diverse learning needs. Female teachers were associated with higher student emotional well-being, consistent with literature suggesting that female teachers often display higher levels of emotional support [66]. Notably, fewer years of teaching experience predicted greater student emotional well-being, potentially due to reduced burnout or increased enthusiasm among early-career teachers. Teacher burnout, which tends to increase with years of service, has been linked to decreased emotional support and may negatively impact both teacher and student well-being [1,67].

Finally, female students demonstrated a higher end-of-school-year GPA than male students, consistent with national trends showing higher academic performance among girls [49,68]. These findings underscore the importance of considering both teacher and student characteristics when evaluating how teacher personality influences developmental outcomes in students with SLDs. Building on the attachment theory, our results suggest that teacher personality traits, particularly openness, neuroticism, and conscientiousness, may shape the emotional and academic outcomes of students with SLDs by influencing how secure, supported, and understood students feel in the classroom [17,18,19,20,21,22].

### 4.2. Implications

The findings of this study offer meaningful implications for educational practice, particularly for supporting the success of students with SLDs. First, the observed associations between teacher personality traits and student outcomes highlight the importance of considering personality dimensions in teacher recruitment, training, and ongoing professional development [23]. Teacher openness reflects qualities such as adaptability, creativity, and openness to experience, all of which are traits that may enhance a teacher’s ability to differentiate instruction and respond to diverse learners’ needs [23,24,61,63]. Integrating strategies that cultivate these qualities in professional learning settings may improve student academic outcomes, especially in inclusive classrooms.

The unexpected positive association between teacher neuroticism and student emotional well-being suggests that emotionally sensitive teachers might create more validating and emotionally attuned environments, particularly beneficial for students with SLDs who may be at greater risk for emotional challenges. Teacher preparation programs may benefit from helping educators channel emotional sensitivity into supportive classroom practices [23,24,25] while also ensuring appropriate emotional regulation strategies are in place to protect teacher well-being [62].

Additionally, the association between teacher conscientiousness and greater student emotional well-being in exploratory analyses suggests that structured, responsible, and dependable teacher behaviors may foster emotional safety and predictability in the classroom, all of which are key components of well-being for students requiring additional support [1,23,24,25,69]. This highlights the potential value of embedding classroom management, organization, and relationship-building skills within teacher development programs.

Organizations have long used personality assessments to guide personnel decisions [70]. However, the findings from the present study suggest that teacher personality traits may not consistently predict student outcomes, particularly for students with SLDs. While certain traits, such as openness and conscientiousness, showed positive associations with student outcomes, others, like agreeableness and extraversion, did not. The unexpected positive link between teacher neuroticism and student emotional well-being further underscores the complexity of teacher–student interactions and cautions against overly simplistic interpretations of personality effects in the classroom.

Rather than relying solely on personality profiles, teacher selection and training processes might benefit more from emphasizing adaptive interpersonal and instructional behaviors [23,62]. For example, traits commonly associated with female teachers, such as empathy and emotional responsiveness [66], were indirectly supported by findings showing that students of female teachers reported higher emotional well-being. These qualities could be more effectively cultivated through targeted professional development than predicted through trait-based assessments alone.

Beyond personality, the findings that female teachers and less experienced teachers were associated with higher student emotional well-being raise important considerations for mentoring and workplace support. It may be that newer teachers, despite having less classroom experience, exhibit higher enthusiasm or lower levels of burnout, which benefits student mental health [71,72,73]. Schools might consider supporting mid- and late-career teachers with emotional wellness initiatives or job re-engagement strategies to mitigate burnout and sustain effective emotional support over time.

Finally, the positive effect of participation in the MindUP program reinforces the value of implementing structured mindfulness-based SEL interventions in middle and high school settings [64,65]. Given the unique academic and emotional needs of students with SLDs, embedding SEL into standard curricula, especially when delivered by emotionally attuned and adaptable teachers, may optimize both academic and psychosocial outcomes. Taken together, these findings point to the need for a more comprehensive approach to teacher development—one that integrates relational competencies, classroom adaptability, and evidence-based intervention delivery to better support students with neurodevelopmental disorders.

### 4.3. Limitations and Future Directions

Several limitations should be considered when interpreting the findings of this study. First, the relatively small sample size, particularly at the teacher level, may have limited statistical power to detect smaller effects and increased the potential for over- or underestimating associations. To address this limitation, we employed a two-level multilevel modeling design, limited the number of predictors to theoretically grounded variables to reduce the risk of overfitting, reported effect sizes (standardized beta coefficients) alongside 95% confidence intervals, and used FIML with the MLR estimator to handle missing data. Although multilevel modeling accounted for the nested data structure, the modest number of clusters (i.e., teachers) may constrain the generalizability and stability of the estimates. Future research should aim to replicate these findings using larger samples across multiple schools or districts to enhance both statistical power and external validity.

One key limitation in the present study was the absence of baseline GPA data. As a result, the multilevel model predicting end-of-school-year academic achievement could not control for students’ prior academic performance. This prevents making any causal inferences about the impact of teacher characteristics on student academic achievement. Future studies should incorporate pre-intervention academic data to better assess change over time.

Additionally, the teachers included in this study taught very different academic subjects at the school, such as math and reading. Considering that SLDs can be specific to a single academic domain, it is possible that the influence of teacher personality may differ depending on subject area and the particular domain of student difficulty. Future research may consider how content-area instruction (e.g., reading vs. math) interacts with teacher traits to shape academic or emotional outcomes in students with SLDs.

Another limitation involves how students were assigned to teachers. Students had different teachers for core subjects such as math, English, and science. Although all teachers delivered the same SEL curriculum (MindUP or NIR), the teacher who completed the personality assessment was not directly tied to the specific subject grades used to calculate each student’s GPA. As a result, multiple teachers contributed to each student’s GPA, but this study focused only on SEL teachers. This creates some uncertainty when interpreting the finding that teacher personality predicted student achievement, since the measured teacher may have influenced only a portion of the student’s GPA. Future research should aim to more directly link teacher characteristics to the specific subjects and outcomes they influence.

This study also relied on a brief self-report personality inventory (BFI-10) [53], which includes only two items per trait and showed limited internal consistency for several subscales in our small sample. The BFI-10 was selected due to its widespread use and practical advantages in survey research, particularly in contexts with limited assessment time [53,55,74,75]. Indeed, the BFI-10 has demonstrated acceptable psychometric properties in large, population-based studies across different countries [55,75,76]. However, consistent with prior cautions in the literature, very brief scales may show attenuated reliability, especially in small samples, and may not fully capture the breadth of each personality domain [77]. Thus, the limited reliability observed in this study may be due in part to the short scale length and small teacher sample size, potentially introducing measurement error. Future research would benefit from using longer, more psychometrically robust personality assessments when feasible to enhance measurement precision and construct validity.

Lastly, the lack of direct assessment of teaching practices and teacher–student relationship quality limits understanding of the mechanisms linking teacher personality to student outcomes. Including such variables in future studies would help clarify these pathways. Despite the above limitations, this study offers valuable insights into how teacher characteristics relate to emotional well-being and academic achievement among students with SLDs. Continued investigation in this area can inform teacher preparation programs, classroom interventions, and inclusive education practices aimed at promoting well-being and success among students with neurodevelopmental disorders.

## 5. Conclusions

This study contributes to the growing body of research on teacher influences in inclusive education by examining how teacher personality traits relate to academic and emotional outcomes among students with SLDs. Using a multilevel framework, the findings revealed that teacher neuroticism and conscientiousness were associated with greater student emotional well-being, while teacher openness was associated with higher student GPAs. The findings highlight the importance of considering teacher characteristics in designing school-based interventions to support the development of learners with SLDs or other neurodevelopmental disorders. In addition to teacher traits, student and teacher demographic variables such as sex, teaching experience, and participation in a mindfulness-based SEL intervention also emerged as important contributors to student outcomes. These results underscore the multifaceted nature of classroom dynamics and the need to consider both interpersonal and contextual factors in supporting the success of students with SLDs. Although limitations related to measurement reliability and sample size warrant caution, the findings offer meaningful implications for teacher training, intervention implementation, and inclusive education policy. These findings contribute to the broader literature on children’s well-being and mental health in educational contexts, emphasizing the importance of teacher characteristics in designing school-based strategies that support the holistic development of students with neurodevelopmental disorders.

## Figures and Tables

**Table 1:** Descriptive statistics and correlations for study variables.

Variable	*n*	*M*	*SD*	Range	1	2	3	4	5	6	7	8	9	10	11	12	13
**Student**																	
1. Age	64	13.28	2.24	10–19	-												
2. Sex	64	0.50	0.50	0–1	−0.03	-											
3. Group	64	0.47	0.50	0–1	−0.05	−0.06	-										
4. Baseline EWB	63	4.11	0.72	2–5	−0.37 **	−0.06	−0.06	-									
5. Post EWB	58	4.08	0.77	2.17–5	−0.38 **	−0.12	−0.08	0.78 **	-								
6. Student GPA	64	3.70	0.35	2.8–4.0	−0.21	0.16	−0.06	−0.07	−0.15	-							
**Teacher**																	
7. Sex	21	0.67	0.48	0–1	−0.49 **	0.07	−0.38 **	0.26 *	0.40 **	0.08	-						
8. Teaching experience	21	7.81	2.89	1–10	−0.19	0.09	0.15	0.04	0.09	0.02	0.35	-					
9. Extraversion	21	2.52	1.03	1–5	0.14	−0.13	0.03	−0.12	−0.16	0.03	−0.13	−0.47 *	-				
10. Agreeableness	21	3.24	0.87	1.5–4.5	−0.44 **	−0.23	−0.09	0.16	0.20	−0.05	0.26	−0.16	0.29	-			
11. Conscientiousness	21	3.93	0.75	2.5–5	−0.04	−0.16	−0.05	0.05	0.19	−0.14	0.00	0.16	−0.14	0.31	-		
12. Neuroticism	21	3.38	0.91	2–5	−0.24	0.04	−0.07	0.24	0.24	0.04	0.02	0.25	0.02	−0.20	−0.01	-	
13. Openness	21	3.83	0.93	2–5	0.02	−0.10	0.32 *	−0.11	−0.25	0.22	−0.47 *	−0.24	0.28	−0.41	−0.45 *	0.14	-

*Note*. EWB = emotional well-being; GPA = grade point average; post = post-intervention. Sex was coded as 0 = male, 1 = female. Group was coded as 0 = Normal Isn’t Real, 1 = MindUP. * *p* < 0.05. ** *p* < 0.01.

**Table 2:** Multilevel model predicting post-intervention emotional well-being.

Predictor	*b*	*SE*	95% CI for *b*	*β*	*p*
**Level 1 (Student Level)**					
Age	0.03	0.08	[–0.12, 0.18]	0.04	0.708
Sex	–0.02	0.12	[–0.25, 0.21]	–0.02	0.860
Intervention Group	0.33	0.16	[0.03, 0.64]	0.26	0.034
Baseline EWB	0.70	0.10	[0.50, 0.90]	0.65	<0.001
**Level 2 (Teacher Level)**					
Sex	0.89	0.19	[0.53, 1.25]	0.83	<0.001
Teaching Experience	–0.09	0.03	[–0.15, –0.04]	–0.52	0.001
Extraversion	–0.15	0.09	[–0.33, 0.04]	–0.29	0.114
Agreeableness	0.03	0.05	[–0.06, 0.13]	0.06	0.478
Conscientiousness	0.28	0.14	[–0.01, 0.56]	0.40	0.055
Neuroticism	0.25	0.08	[0.09, 0.42]	0.44	0.003
Openness	–0.04	0.10	[–0.24, 0.16]	–0.07	0.701

*Note*. EWB = emotional well-being; *b* = unstandardized regression coefficient; *SE* = standard error; CI = confidence interval; *β* = standardized coefficient; *p* = significance value. Student-level predictors were group-mean centered; teacher-level predictors were grand-mean centered. Sex was coded as 0 = male, 1 = female. Intervention group was coded as 0 = Normal Isn’t Real, 1 = MindUP. *n* = 57 students with complete data.

**Table 3:** Multilevel model predicting end-of-school-year GPA.

Predictor	*b*	*SE*	95% CI for *b*	*β*	*p*
**Level 1 (Student-Level)**					
Age	–0.02	0.05	[–0.11, 0.08]	–0.04	0.772
Sex	0.14	0.07	[0.01, 0.28]	0.21	0.038
Intervention Group	–0.10	0.09	[–0.26, 0.07]	–0.14	0.256
**Level 2 (Teacher-Level)**					
Sex	0.09	0.11	[–0.12, 0.30]	0.37	0.393
Teaching Experience	0.01	0.02	[–0.02, 0.04]	0.24	0.560
Extraversion	–0.01	0.06	[–0.12, 0.11]	–0.05	0.923
Agreeableness	0.05	0.08	[–0.10, 0.20]	0.37	0.498
Conscientiousness	0.02	0.07	[–0.11, 0.15]	0.14	0.731
Neuroticism	–0.02	0.04	[–0.10, 0.07]	–0.11	0.736
Openness	0.17	0.07	[0.03, 0.31]	1.29	0.022

*Note*. GPA = grade point average; *b* = unstandardized regression coefficient; *SE* = standard error; CI = confidence interval; *β* = standardized coefficient; *p* = significance value. Student-level predictors were group-mean centered; teacher-level predictors were grand-mean centered. Sex was coded as 0 = male, 1 = female. Intervention group was coded as 0 = Normal Isn’t Real, 1 = MindUP. *n* = 64 students with complete data.

## Data Availability

The data presented in this study are available on request from the corresponding author due to privacy reasons.

## References

[1] Jennings P.A., Greenberg M.T. (2009). The prosocial classroom: Teacher social and emotional competence in relation to student and classroom outcomes. Rev. Educ. Res..

[2] Zheng F. (2022). Fostering students’ well-being: The mediating role of teacher interpersonal behavior and student-teacher relationships. Front. Psychol..

[3] Bonuomo M., Marini M., Vegni N., Melogno S., Torregiani G., Livi S., Di Filippo G. (2023). Analysis of psychological and social functioning in undergraduate students with a specific learning disorder (SLD). Brain Sci..

[4] Matteucci M.C., Scalone L., Tomasetto C., Cavrini G., Selleri P. (2019). Health-related quality of life and psychological wellbeing of children with specific learning disorders and their mothers. Res. Dev. Disabil..

[5] American Psychiatric Association (2022). Diagnostic and Statistical Manual of Mental Disorders.

[6] Grigorenko E.L., Compton D.L., Fuchs L.S., Wagner R.K., Willcutt E.G., Fletcher J.M. (2020). Understanding, educating, and supporting children with specific learning disabilities: 50 years of science and practice. Am. Psychol..

[7] Piko B.F., Dudok R. (2023). Strengths and difficulties among adolescent with and without specific learning disorders (SLD). Children.

[8] Mugnaini D., Lassi S., La Malfa G., Albertini G. (2009). Internalizing correlates of dyslexia. World J. Pediatr..

[9] Visser L., Kalmar J., Linkersdörfer J., Görgen R., Rothe J., Hasselhorn M., Schulte-Körne G. (2020). Comorbidities between specific learning disorders and psychopathology in elementary school children in Germany. Front. Psychiatry.

[10] Carroll J.M., Maughan B., Goodman R., Meltzer H. (2005). Literacy difficulties and psychiatric disorders: Evidence for comorbidity. J. Child Psychol. Psychiatry.

[11] Francis D.A., Caruana N., Hudson J.L., McArthur G.M. (2019). The association between poor reading and internalising problems: A systematic review and meta-analysis. Clin. Psychol. Rev..

[12] Hendren R.L., Haft S.L., Black J.M., White N.C., Hoeft F. (2018). Recognizing psychiatric comorbidity with reading disorders. Front. Psychiatry.

[13] Cortiella C., Horowitz S.H. (2014). The State of Learning Disabilities: Facts, Trends and Emerging Issues.

[14] Orr A.C., Goodman N. (2010). “People like me don’t go to college:” The legacy of learning disability. J. Ethnogr. Qual. Res..

[15] Stein B., Hoeft F., Richter C.G. (2024). Stress, resilience, and emotional well-being in children and adolescents with specific learning disabilities. Curr. Opin. Behav. Sci..

[16] Catts H.W., Petscher Y. (2021). A cumulative risk and resilience model of dyslexia. J. Learn. Disabil..

[17] Bowlby J. (1958). The nature of the child’s tie to his mother. Int. J. Psychoanal..

[18] Verschueren K., Koomen H.M.Y. (2012). Teacher–child relationships from an attachment perspective. Attach. Hum. Dev..

[19] García-Rodríguez I., Carracedo C.H., Suárez Á.L., González-Castro P., Álvarez-García D. (2023). Teacher–student attachment relationships and academic and emotional outcomes in children: A systematic review. Educ. Res. Rev..

[20] Li X., Duan S., Liu H. (2023). Unveiling the predictive effect of students’ perceived EFL teacher support on academic achievement: The mediating role of academic buoyancy. Sustainability.

[21] He L., Feng L., Ding J. (2024). The relationship between perceived teacher emotional support, online academic burnout, academic self-efficacy, and online English academic engagement of Chinese EFL learners. Sustainability.

[22] Huang L., Wang D. (2023). Teacher support, academic self-efficacy, student engagement, and academic achievement in emergency online learning. Behav. Sci..

[23] Göncz L. (2017). Teacher personality: A review of psychological research and guidelines for a more comprehensive theory in educational psychology. Open Rev. Educ. Res..

[24] Kim L.E., Jörg V., Klassen R.M. (2019). A meta-analysis of the effects of teacher personality on teacher effectiveness and burnout. Educ. Psychol. Rev..

[25] Perera H.N., McIlveen P. (2018). Profiles of teacher personality and relationships with teacher self-efficacy, work engagement, and job satisfaction. Pers. Individ. Dif..

[26] Zee M., Koomen H.M.Y. (2016). Teacher self-efficacy and its effects on classroom processes, student academic adjustment, and teacher well-being: A synthesis of 40 years of research. Rev. Educ. Res..

[27] Brännlund A., Strandh M., Nilsson K. (2017). Mental-health and educational achievement: The link between poor mental-health and upper secondary school completion and grades. J. Ment. Health.

[28] McLeod J.D., Kaiser K. (2004). Childhood emotional and behavioral problems and educational attainment. Am. Sociol. Rev..

[29] Haft S.L., Myers C.A., Hoeft F. (2016). Socio-emotional and cognitive resilience in children with reading disabilities. Curr. Opin. Behav. Sci..

[30] Al-Yagon M. (2016). Perceived close relationships with parents, teachers, and peers: Predictors of social, emotional, and behavioral features in adolescents with LD or comorbid LD and ADHD. J. Learn. Disabil..

[31] Kiuru N., Poikkeus A.-M., Lerkkanen M.-K., Pakarinen E., Siekkinen M., Ahonen T., Nurmi J.-E. (2012). Teacher-perceived supportive classroom climate protects against detrimental impact of reading disability risk on peer rejection. Learn. Instr..

[32] Baker J.A., Grant S., Morlock L. (2008). The teacher-student relationship as a developmental context for children with internalizing or externalizing behavior problems. Sch. Psychol. Q..

[33] Danielsen A.G., Samdal O., Hetland J., Wold B. (2010). School-related social support and students’ perceived life satisfaction. J. Educ. Res..

[34] Furrer C.J., Skinner E.A., Pitzer J.R. (2014). The influence of teacher and peer relationships on students’ classroom engagement and everyday motivational resilience. Teach. Coll. Rec..

[35] Hamre B.K., Pianta R.C. (2003). Early teacher-child relationships and the trajectory of children’s school outcomes through eighth grade. Child Dev..

[36] Kashy-Rosenbaum G., Kaplan O., Israel-Cohen Y. (2018). Predicting academic achievement by class-level emotions and perceived homeroom teachers’ emotional support. Psychol. Sch..

[37] Wang M.-T., Degol J.L., Amemiya J., Parr A., Guo J. (2020). Classroom climate and children’s academic and psychological well-being: A systematic review and meta-analysis. Dev. Rev..

[38] Yang Y., Li G., Su Z., Yuan Y. (2021). Teacher’s emotional support and math performance: The chain mediating effect of academic self-efficacy and math behavioral engagement. Front. Psychol..

[39] Roorda D.L., Koomen H.M.Y., Spilt J.L., Oort F.J. (2011). The influence of affective teacher–student relationships on students’ school engagement and achievement: A meta-analytic approach. Rev. Educ. Res..

[40] McCormick M.P., O’Connor E.E., Cappella E., McClowry S.G. (2013). Teacher–child relationships and academic achievement: A multilevel propensity score model approach. J. Sch. Psychol..

[41] Mattison R.E., Woods A.D., Morgan P.L., Farkas G., Hillemeier M.M. (2022). Longitudinal trajectories of reading and mathematics achievement for students with learning disabilities. J. Learn. Disabil..

[42] Sideridis G.D. (2007). International approaches to learning disabilities: More alike or more different?. Learn. Disabil. Res. Pract..

[43] Eilam B., Vidergor H.E. (2011). Gifted Israeli students’ perceptions of teachers’ desired characteristics: A case of cultural orientation. Roeper Rev..

[44] Eryilmaz A. (2014). Perceived personality traits and types of teachers and their relationship to the subjective well-being and academic achievements of adolescents. Educ. Sci. Theory Pract..

[45] Goldstein G.S., Benassi V.A. (2006). Students’ and instructors’ beliefs about excellent lecturers and discussion leaders. Res. High. Educ..

[46] Kell H.J. (2019). Do teachers’ personality traits predict their performance? A comprehensive review of the empirical literature from 1990 to 2018. ETS Res. Rep. Ser..

[47] Nkomo N.N., Ochiche U.P., Akpan E.M. (2022). The influence of teachers’ agreeableness and openness to experience on secondary school students’ English language academic achievement in Ogoja Education Zone of Cross River State, Nigeria. J. Educ. Pract..

[48] Kim L.E., Dar-Nimrod I., MacCann C. (2018). Teacher personality and teacher effectiveness in secondary school: Personality predicts teacher support and student self-efficacy but not academic achievement. J. Educ. Psychol..

[49] Hwang N., Fitzpatrick B. (2021). Student–teacher gender matching and academic achievement. AERA Open.

[50] Kini T., Podolsky A. (2016). Does teaching experience increase teacher effectiveness? A review of the research. Learn. Policy Inst..

[51] Maloney J.E., Lawlor M.S., Schonert-Reichl K.A., Whitehead J. (2016). A mindfulness-based social and emotional learning curriculum for school-aged children: The MindUP program. Handbook of Mindfulness in Education: Integrating Theory and Research into Practice.

[52] Banerjee M., Lalor A.R. (2021). Toolkit for Normal Isn’t Real: Succeeding with Learning Disabilities & ADHD.

[53] Rammstedt B., John O.P. (2007). Measuring personality in one minute or less: A 10-item short version of the Big Five Inventory in English and German. J. Res. Pers..

[54] Eisinga R., Te Grotenhuis M., Pelzer B. (2012). The reliability of a two-item scale: Pearson, Cronbach, or Spearman-Brown?. Int. J. Public Health.

[55] Costa Mastrascusa R., de Oliveira Fenili Antunes M.L., de Albuquerque N.S., Virissimo S.L., Foletto Moura M., Vieira Marques Motta B., de Lara Machado W., Moret-Tatay C., Quarti Irigaray T. (2023). Evaluating the complete (44-item), short (20-item) and ultra-short (10-item) versions of the Big Five Inventory (BFI) in the Brazilian population. Sci. Rep..

[56] Bickman L., Athay M.M., Riemer M., Lambert E.W., Kelley S.D., Breda C., Tempesti T., Dew-Reeves S.E., Brannan A.M., Vides de Andrade A.R. (2010). Manual of the Peabody Treatment Progress Battery.

[57] Athay M.M., Kelley S.D., Dew-Reeves S.E. (2012). Brief multidimensional students’ life satisfaction scale—PTPB version (BMSLSS-PTPB): Psychometric properties and relationship with mental health symptom severity over time. Adm. Policy Ment. Health Ment. Health Serv. Res..

[58] Huebner E.S., Seligson J.L., Valois R.F., Suldo S.M. (2006). A review of the Brief Multidimensional Students’ Life Satisfaction Scale. Soc. Indic. Res..

[59] Hedges L.V., Hedberg E.C. (2007). Intraclass correlation values for planning group-randomized trials in education. Educ. Eval. Policy Anal..

[60] McLean L., Connor C.M. (2015). Depressive symptoms in third-grade teachers: Relations to classroom quality and student achievement. Child Dev..

[61] John O.P., Srivastava S., Pervin L.A., John O.P. (1999). The Big Five trait taxonomy: History, measurement, and theoretical perspectives. Handbook of Personality: Theory and Research.

[62] Roeser R.W., Skinner E., Beers J., Jennings P.A. (2012). Mindfulness training and teachers’ professional development: An emerging area of research and practice. Child Dev. Perspect..

[63] Decker D.M., Rimm-Kaufman S.E. (2008). Personality characteristics and teacher beliefs among pre-service teachers. Teach. Educ. Q..

[64] Schonert-Reichl K.A., Oberle E., Lawlor M.S., Abbott D., Thomson K., Oberlander T.F., Diamond A. (2015). Enhancing cognitive and social–emotional development through a simple-to-administer mindfulness-based school program for elementary school children: A randomized controlled trial. Dev. Psychol..

[65] Taylor R.D., Oberle E., Durlak J.A., Weissberg R.P. (2017). Promoting positive youth development through school-based social and emotional learning interventions: A meta-analysis of follow-up effects. Child Dev..

[66] Graziano F., Mastrokoukou S., Monchietto A., Marchisio C., Calandri E. (2024). The moderating role of emotional self-efficacy and gender in teacher empathy and inclusive education. Sci. Rep..

[67] Chang M.L. (2009). An appraisal perspective of teacher burnout: Examining the emotional work of teachers. Educ. Psychol. Rev..

[68] Duckworth A.L., Seligman M.E.P. (2006). Self-discipline gives girls the edge: Gender in self-discipline, grades, and achievement test scores. J. Educ. Psychol..

[69] Iznardo M., Ryan J., Rogers M., McKibbin S., Piers L., Hogan T. (2023). Examining resiliency in children with learning disabilities and co-occurring ADHD symptoms: The protective role of a close teacher-student relationship. Learn. Disabil. Contemp. J..

[70] Salgado J.F., De Fruyt F., Evers A., Anderson N., Voskuijl O. (2017). Personality in personnel selection. The Blackwell Handbook of Personnel Selection.

[71] Harding S., Morris R., Gunnell D., Ford T., Hollingworth W., Tilling K., Evans R., Bell S., Grey J., Brockman R. (2019). Is teachers’ mental health and wellbeing associated with students’ mental health and wellbeing?. J. Affect. Disord..

[72] Madigan D.J., Kim L.E. (2021). Towards an understanding of teacher attrition: A meta-analysis of burnout, job satisfaction, and teachers’ intentions to quit. Teach. Teach. Educ..

[73] Oberle E., Schonert-Reichl K.A. (2016). Stress contagion in the classroom? The link between classroom teacher burnout and morning cortisol in elementary school students. Soc. Sci. Med..

[74] Gaisendrees L., John O.P., Rammstedt B. (2022). Measuring the Big Five with single items using the BFI-S: A replication and extension. Adv. Open Educ..

[75] Rammstedt B., Kemper C.J., Klein M.C., Beierlein C., Kovaleva A. (2013). Eine kurze Skala zur Messung der fünf Dimensionen der Persönlichkeit: BFI-10 [A short scale for assessing the Big Five dimensions of personality: BFI-10]. Methods Data Anal..

[76] Courtois R., Petot J.-M., Plaisant O., Allibe B., Lignier B., Réveillère C. (2020). Validation française du Big Five Inventory à 10 items (BFI-10) [French validation of the Big Five Inventory–10 (BFI-10)]. L’Encéphale.

[77] Chapman B.P., Elliot A.J. (2017). Brief report: How short is too short? An ultra-brief measure of the big-five personality domains implicates “agreeableness” as a risk for all-cause mortality. J. Health Psychol..

